# Mysteries of adenovirus packaging

**DOI:** 10.1128/jvi.00180-25

**Published:** 2025-04-17

**Authors:** Matthew Charman, Matthew D. Weitzman

**Affiliations:** 1 Division of Protective Immunity and Division of Cancer Pathobiology, The Children’s Hospital of Philadelphia, Philadelphia, Pennsylvania, USA; 2 Department of Pathology and Laboratory Medicine, University of Pennsylvania Perelman School of Medicine 14640 , Philadelphia, Pennsylvania, USA; 3 Penn Epigenetics Institute, University of Pennsylvania Perelman School of Medicine 14640 , Philadelphia, Pennsylvania, USA; 4 Penn Center for Genome Integrity, University of Pennsylvania Perelman School of Medicine 14640 , Philadelphia, Pennsylvania, USA; New York University Department of Microbiology, New York, New York, USA

**Keywords:** adenoviruses, genome packaging, capsid assembly, delivery vectors

## Abstract

It is conventionally held that most DNA viruses package their genomes by one of two fundamental mechanisms: described by the sequential or concurrent models of assembly and packaging. Sequential packaging involves the translocation of a viral genome into a pre-formed capsid, often referred to as the pro-capsid. In contrast, concurrent packaging does not require the assembly of a pro-capsid. Instead, the genome is condensed, and the capsid shell is formed around the genome. The accumulation of empty particles in adenovirus infected cells has led to the assumption that adenovirus packaging may be best described by the sequential model. However, existing models fail to adequately explain all experimental observations, leaving many mysteries of adenovirus genome packaging unresolved. In this review, we describe key findings in adenovirus assembly and packaging, and we discuss them in the context of the competing models of sequential versus concurrent packaging. We discuss recent findings that have redefined our understanding of adenovirus packaging, including the role of viral biomolecular condensates and visualization of viral assembly and packaging *in situ*. These advances have renewed interest in the concurrent model of packaging. We anticipate that lessons learned from adenovirus packaging will be highly valuable for the advancement of viral vectors and gene-delivery technologies. In reviewing this topic, we hope to stimulate discussion and facilitate future investigation that will ultimately resolve gaps in knowledge and expand our understanding of DNA virus genome packaging.

## INTRODUCTION TO DNA VIRUS PACKAGING

DNA viruses include a diverse range of important human pathogens and powerful therapeutic tools. These agents are obligate intracellular parasites that can only replicate inside the living cells of a host organism. Outside of host cells, they exist as viral particles. Viral particles act as gene delivery vehicles, protecting viral genomes from their environment, facilitating the spread of infection between cells, and enabling transmission between hosts. Successful delivery of the viral genome into host cells and evasion of intracellular defenses enable a program of viral gene expression that hijacks the host cell, enables viral DNA replication, and ultimately leads to the packaging of DNA genomes into new progeny particles (1–4). Many DNA viruses can package and deliver their genomes with exceptional efficiency. This has led to the repurposing of certain DNA viruses as gene delivery vectors that find use in gene therapy, oncolytic therapy, and vaccination (5–11). However, natural restrictions on coding capacity, customization, and production methods limit the therapeutic use of viral vectors (5–11). The development of next-generation gene-delivery vectors that circumvent these limitations will likely require an advanced understanding of viral genome packaging, necessitating further research into this important viral process.

**Fig 1:**
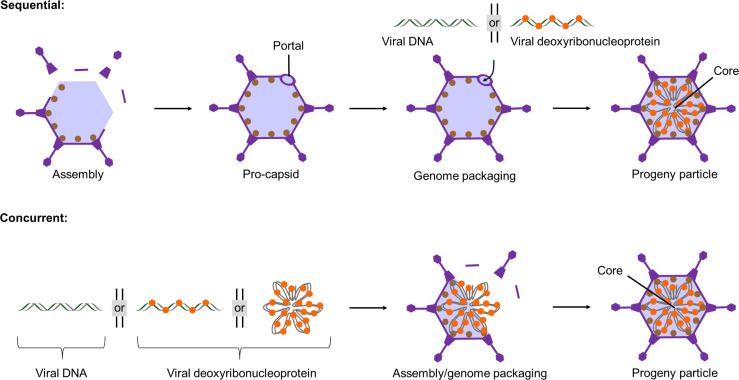
Simplified schematic of proposed assembly and packaging mechanisms of DNA viruses. Assembly and packaging of most DNA viruses can be described by two fundamental models, sequential or concurrent. Viral DNA genomes may be single-stranded or double-stranded and may take different forms including circular or linear. For simplicity's sake, linear double-stranded DNA is shown. Sequential model: The capsid shell (pro-capsid) is first assembled and then subsequently packaged by translocation of the viral genome through the portal. The viral genome can be packaged as naked DNA or as a deoxyribonucleoprotein complex. The example shown is of a progeny particle containing a deoxyribonucleoprotein core. Concurrent model: The viral genome acts as a substrate around which the capsid assembles.

It is proposed that packaging of viral DNA genomes typically occurs via one of the two fundamental mechanisms as described by the sequential or concurrent models of assembly and packaging (12–17). The sequential packaging mechanism is common among larger DNA viruses and is exemplified by certain bacteriophages and the prototypic herpesvirus herpes simplex virus 1 (HSV-1) (12, 15, 18). Sequential packaging involves the translocation of a viral genome into a pre-formed capsid, often referred to as the pro-capsid (Fig. 1A). The packaging machinery of prototype viruses has been investigated in detail, and high-resolution structural data have guided our understanding of the mechanisms by which these molecular machines pump the viral genome into the procapsid (19–21). In contrast, concurrent packaging does not require the assembly of a pro-capsid. Instead, the capsid shell is assembled around the genome (Fig. 1B). The genome may be encapsidated as a nucleoprotein complex that comprises the genome in association with viral nucleic acid binding proteins, sometimes referred to as the pre-core. Viruses that package progeny particles via the concurrent mechanism, such as members of the polyomaviruses and papillomaviruses, do not require a portal or portal complex and are typically smaller and simpler than viruses that assemble and package sequentially (22, 23). Despite being a central feature for viruses as both pathogens and gene delivery tools, many mysteries of viral assembly and packaging remain. Indeed, our understanding of viral assembly and packaging relies heavily on extrapolation from a relatively small number of DNA viruses, for which these processes have been well-characterized. In some cases, broader models of genome packaging do not adequately explain all experimental observations. This suggests that existing models may fail to capture the full complexity of DNA packaging and that crucial gaps in knowledge remain. This point is well illustrated by adenovirus (AdV). AdV has been extensively studied over several decades, providing crucial insight into many fundamental viral and host processes (24–32). However, recent reports suggest that we may be further from understanding the true complexity of AdV packaging than previously appreciated.

## ARE ADENOVIRUS CAPSID ASSEMBLY AND GENOME PACKAGING SEQUENTIAL OR CONCURRENT PROCESSES?

Human adenoviruses comprise seven species of the Mastadenovirus genus (designated A–G), including over 50 distinct serotypes defined by the absence of serological cross-neutralization and over 110 types based on genetic analysis (33, 34). Serotypes 2 and 5, which belong to species C, are the best characterized with regard to their molecular biology and have been commonly used to study viral processes such as assembly and packaging. AdV is a ubiquitous and clinically important respiratory and gastrointestinal pathogen that causes mild-to-severe disease in infected individuals (35–38). Prolific in its ability to replicate and transmit, the CDC reports AdV as the most common cause of viral outbreaks in the USA, with concerns over pandemic potential increasing (39–41). AdV is also a highly valuable therapeutic tool that has been adapted for use in cancer therapy, vaccination, and gene therapy (5–9, 42–45).

Over the last few decades, significant progress has been made in understanding the molecular biology of AdV infection, including insights into AdV particle assembly. The key viral proteins required for assembly and packaging have been discovered, and the composition and structure of viral particles have been defined in great detail (46–52). However, some fundamental gaps in knowledge remain. For example, recent findings suggest that the field has still yet to resolve whether AdV uses a sequential or concurrent packaging mechanism. These gaps in knowledge may hamper attempts to improve strategies that target or repurpose packaging for therapeutic gain. In this review, we revisit AdV genome packaging. We describe key findings in particle assembly and genome packaging and discuss these in the context of the sequential versus concurrent assembly models. In doing so, we hope to stimulate discussion and facilitate future investigation that will ultimately resolve gaps in knowledge and progress our understanding of viral genome packaging.

## THE ADENOVIRUS REPLICATIVE CYCLE

The AdV particle is a non-enveloped icosahedron approximately 100 nm in diameter (47, 53). Each icosahedral progeny particle comprises an outer capsid shell made up of the viral major capsid proteins hexon, penton base, and fiber. Minor capsid protein IX is also present in the capsid exterior, functioning as a cement that helps stabilize the capsid (47, 54, 55). Within the inner cavity of the capsid shell, the viral core comprises core proteins V, VII, μ, and the virus-encoded L3-23 kilodalton protease (often referred to simply as the adenovirus protease or AVP), in association with the condensed viral genome (56, 57). The viral terminal protein (TP), which functions in the replication of the dsDNA genome, is also present in the core, as it is covalently attached to the viral genome (58, 59). Minor capsid proteins IIIa, VI, and VIII are present in the capsid’s interior surface and stabilize the capsid and link the inner core and capsid shell (48, 55–57, 60). Viral core and cement proteins cooperate in tethering the viral genome to the capsid and stabilizing the viral particle (47, 48, 57, 60–62).

Following attachment and uptake by host cells, viral particles undergo a process of controlled disassembly, resulting in endosomal escape and release of the viral genome for import into the nucleus (51, 63–74). Exposure of core protein VI plays an important role in the escape of the particle from the endosome, likely by disrupting the endosomal membrane (56, 65, 75–78). Once inside the nucleus, a program of viral early gene expression is initiated (4, 79, 80). Viral early genes encode proteins that interact with host factors, manipulating the host cell and making it conducive to viral replication (81, 82). Viral early genes also encode the machinery required to replicate the viral genome, which takes place at viral replication compartments (VRCs) (1, 83, 84). In addition to TP, viral genome replication requires the viral DNA-binding protein (DBP) and viral polymerase (58, 85, 86). Host-cell proteins recruited to replicating genomes also contribute to DNA synthesis (58, 85). Viral genome replication is required for activation of the viral major late promoter (MLP) and progression to the late stage of infection (87–90). Activation of the MLP is facilitated by the viral protein IVa2, resulting in the expression of viral late genes. Late gene products include the structural proteins that make up the completed viral particle and packaging proteins essential for encapsidation of the viral genome (49, 58, 91, 92).

Viral late proteins are translated into the cytoplasm and imported into the nucleus where viral capsid assembly and genome packaging take place. Cooperation between viral proteins helps ensure efficient nuclear import of the required late proteins. For example, hexon trimers, the major component of the viral capsid, first assemble from hexon monomers in the cytoplasm and are imported into the nucleus in association with protein VI (93). It has also been suggested that the assembly and import of hexon trimers, as well as their higher-order assembly into capsids, may be aided by a chaperone or scaffolding function provided by the L4-100K protein, since the accumulation of nuclear hexon trimers and viral particles is limited in the absence of functional L4-100K (94–98). A complex combination of interactions between the viral genome, genome-associated core proteins, minor capsid proteins, and the L1-52-55 kilodalton protein (L1-52K) are thought to help tether the viral genome to the internal surface of the capsid during packaging (47–49, 56, 61, 99, 100). Genome packaging leads to particle maturation, which yields infectious progeny particles for spread and transmission (101, 102). Maturation is mediated by the adenovirus protease (101, 103). Protein VI and packaged DNA act as cofactors for AVP, which cleaves precursor forms of the minor capsid (pIIIa, pVI, and pVIII) and core (pVII, pμ, and pTP) proteins (51, 56, 101, 104–106). In addition, AVP cleaves L1-52K, which is removed from the viral particle (51, 107, 108). Maturation must take place either during or after packaging, since incomplete particles lacking packaged genomes contain only precursor forms of AVP substrate proteins (51, 107–111). If the genome is packaged into a pre-formed capsid, then this raises an interesting conundrum. How does L1-52K escape the internal cavity of a completed capsid following packaging and maturation? In contrast, concurrent capsid assembly and genome packaging might allow for the maturation and release of L1-52K before the capsid is completed.

## REQUIREMENTS FOR GENOME PACKAGING: PROTEINS AND Ψ

Genetic approaches have been used to investigate the requirements for genome packaging by generating viral mutants that exhibit loss-of-function or conditional loss-of-function phenotypes (49, 52). Such studies identified several viral proteins required for packaging of the viral genome, as well as the AdV packaging sequence (ψ), present on the left end of the AdV genome close to the left inverted terminal repeat (ITR) (112, 113). The ψ sequence is composed of seven functionally redundant AT-rich repeats with the sequence motif 5′-TTTGN8CG- 3’, numbered A1–A7. There is a 21-nucleotide spacer between repeats A1 and A2 and between repeats A5 and A6, but not between other repeats (113–119). This spacing corresponds to two complete turns of a DNA helix, placing the adjacent repeats two major grooves apart. The importance of ψ is undeniable, as multiple studies have demonstrated that only empty or incomplete particles accumulate in the absence of a functional packaging sequence (113–119). Although only the A1–A2 and A5–A6 repeats are absolutely essential, packaging is most efficient when all seven repeats are present (117, 119). Interestingly, experimental repositioning of ψ demonstrated that packaging can be successfully mediated by ψ present at either the left or right end of the viral genome. In addition, packaging was functional when ψ was placed within 1,000 nucleotides of the left ITR (113, 114). These findings indicate that although packaging requires a ψ sequence near one end of the viral genome, the precise wild-type location is not essential (120).

The presence of an essential packaging sequence led to the suggestion that this sequence may function by providing a binding site for a protein or protein complex involved in genome packaging. Components of the putative AdV packaging complex have been elucidated through multiple lines of investigation, including co-precipitation with viral genomes from infected cell lysates, interaction with other putative packaging factors, and interactions with the packaging sequence *in vitro* (49, 52, 121–126). The packaging complex is thought to comprise the L1-52K, IVa2, IIIa, L4-22 kilodalton (L4-22K), and L4-33 kilodalton (L4-33K) proteins (121, 122). In addition, host proteins such as COUP-TF, Oct-1, and CCAAT displacement protein can also interact with the ψ sequence, although it is unclear whether these transcription factors are required for genome packaging (115). The IVa2 and L1-52K proteins are both associated with the Ψ sequence in AdV-infected cells (121). Although protein IVa2 interacts directly with the packaging sequence *in vitro*, protein L1-52K does not (122). This suggests that IVa2 could play an important role in Ψ recognition. Interestingly, the IVa2 and L1-52K proteins interact directly with each other *in vitro,* suggesting that IVa2 could be responsible for the recruitment of L1-52K to the packaging sequence (125). However, L1-52K interacts with the Ψ sequence in cells infected with pm8002, a mutant virus that does not express the IVa2 protein (122). Similarly, IVa2 interacts with the Ψ sequence in cells infected with pm8001, a mutant virus that does not express the L1-52K protein (122). This suggests that recognition of ψ by the full packaging complex may involve additional protein-DNA and protein-protein interactions. Several studies have demonstrated that functional versions of these proteins are required for the production of packaged progeny particles, reinforcing the designation of L1-52K, IVa2, IIIa, L4-22K, and L4-33K as packaging proteins (108, 124, 127–131).

Collectively, these findings indicate that AdV genome packaging requires a specific viral packaging sequence and packaging proteins. However, these requirements do not clarify the fundamental mechanisms by which AdV genomes are packaged, since a packaging sequence and dedicated packaging proteins can be a feature of both sequential and concurrent packaging models.

## GENOME PACKAGING REQUIRES ACTIVE GENOME REPLICATION

An interesting feature of AdV packaging is the requirement for active genome replication. Many pioneering studies that sought to identify key determinants of viral multiplication utilized temperature-sensitive mutants isolated following viral replication in the presence of chemical mutagens (132). Temperature-sensitive mutant viruses are phenotypically wild-type when infection is allowed to proceed at the permissive temperature but exhibit defects at their non-permissive temperatures, allowing for conditional control of viral processes via temperature shift (132). The ts3 mutant, which carries a temperature-sensitive mutation that maps to a viral late gene somewhere in the L2 or L3 region, is defective in virion assembly at its non-permissive temperature but will assemble capsids and package genomes when shifted to the permissive temperature (133, 134). Radiolabeling of viral DNA synthesis in ts3-infected cells indicates that although viral genomes accumulate at the non-permissive temperature, when assembly and packaging are restored, only newly synthesized genomes are packaged. This suggests that packaging requires active genome replication (135). Similarly, mutants with temperature-sensitive defects in DBP allow for controlled inhibition of genome replication. When temperature-sensitive mutant viruses that are defective in DNA replication are shifted to their non-permissive temperature, no packaging takes place, even when the late phase has been reached and sufficient late proteins accumulated (135, 136). In agreement with these observations, inhibiting DNA replication using hydroxyurea also blocks packaging (135). The viral DBP, which is an essential component of the viral DNA replication machinery, interacts with packaging proteins IVa2 and L4-33K and can be detected in some preparations of viral particles purified by CsCl-gradient ultracentrifugation (123, 135, 137–139). Collectively, these findings suggest that replication of the viral genome is coupled with packaging. However, the requirement for active DNA replication alone does not clarify the fundamental packaging mechanism employed by AdV. Sequential packaging of viral genomes into pre-formed capsids can occur in a replication-dependent manner in which genomes are threaded through the portal as they are replicated (12, 18). Similarly, concurrent packaging could also utilize newly replicated genomes and would explain why empty particles assembled during defective packaging do not convert to packaged particles when packaging is restored. Whichever fundamental mechanism is at play, elucidating the molecular details of how genome replication and packaging are coupled will likely provide key insight into the AdV packaging mechanism.

## EMPTY CAPSIDS: ESSENTIAL ASSEMBLY INTERMEDIATES OR DEAD-END BY-PRODUCTS?

Some of the earliest evidence contributing to discussions on the mechanism for AdV assembly and packaging came from the observation that different types of viral particles accumulate in infected cells. It is possible to separate particle types using CsCl-gradient ultracentrifugation of the infected cell lysates, since mature packaged particles are denser than immature or empty particles (61, 92, 109–111, 140). Early experiments revealed that although the majority of particles assembled during wild-type virus infection are mature packaged particles, a smaller proportion of empty capsids and incomplete particles also accumulate (61, 92, 109–111, 140). It was found that true empty capsids lack viral genomes and genome-associated core proteins (92, 111, 141). This observation was confirmed by transmission electron microscopy, with packaged particles identifiable due to the greater electron density of the viral core (92, 127, 142). In contrast, incomplete particles typically co-purify with viral genomes and contain precursor forms of core proteins indicative of particles that have either started but not completed genome packaging or have completed packaging but not maturation. Interestingly, these incomplete particles are preferentially associated with the left end of the genome, suggesting that packaging is initiated at the packaging sequence (108, 118, 143, 144). Although empty and incomplete particles co-sediment with similar coefficients, it is possible to separate these different particle types based on their subtly different densities by employing sequential rounds of purification using high-resolution purification gradients. Indeed, incomplete particles purified from infected cells can also be separated into more than one distinct sub-type of incomplete particle, presumably with different compositions (61). For this reason, the methodology used to purify viral particles should be considered when interpreting particle composition, as viral isolates may be heterotypic.

Empty pro-capsids are essential assembly intermediates for viruses that utilize sequential assembly and packaging mechanisms (12, 15, 18, 19). Accordingly, some have suggested that the empty capsids found in cells infected with AdV must represent pro-capsids, implying that AdV assembly and packaging are sequential processes (49, 92). However, this assertion fails to consider the possibility that the empty particles accumulated during AdV infection are not essential precursors but may instead be dead-end by-products of an imperfect concurrent assembly and packaging process. Concurrent packaging of small DNA virus genomes is exemplified by the assembly and packaging of papillomaviruses and polyomaviruses, which have established a framework for understanding concurrent packaging in DNA viruses (22, 23). Papillomavirus and polyomavirus virions contain viral genomes complexed with cellular histones, termed viral micro-chromosomes, or microsomes (145, 146). Microsomes assemble in the nucleus and are thought to provide a substrate around which the capsid is assembled (22, 23, 145, 146). However, papillomavirus and polyomavirus capsids can also form in the absence of viral genomes. For example, papillomavirus and polyomavirus capsid proteins alone can self-assemble into virus-like particles when expressed in insect cells or when purified capsid proteins are assembled *in vitro* (147–152). In the case of human papillomavirus (HPV), these virus-like particles have been utilized to great effect in subunit vaccines that protect against HPV and HPV-associated cancer (153, 154). It seems plausible, therefore, that empty capsids could accumulate in adenovirus-infected cells due to spontaneous capsid assembly that results from imperfect coordination of events that link capsid assembly and genome condensation. For this reason, the detection of empty capsids in infected cells should not alone be considered sufficient evidence to support a sequential model of capsid assembly and genome packaging. Instead, we must seek to understand better the relationship between different types of viral particles accumulated during infection.

## TIME-RESOLVED ANALYSES OF CAPSID ASSEMBLY, GENOME PACKAGING, AND PARTICLE MATURATION

Various approaches have been combined with pulse-chase labeling of viral proteins and DNA using radiolabeled isotopes in an attempt to establish the temporal relationship between different types of particles accumulated during infection (92, 110, 111, 136, 140, 155–158). During wild-type virus infection, radio-labeled amino acids are incorporated into empty, incomplete, and complete particles rapidly (0–2 h post-addition). As the infection progresses, all types of radiolabeled particles accumulate in parallel (92, 111). These findings suggest that both capsid assembly and genome packaging generally occur throughout the late phase of infection, rather than occurring in sequential, temporally distinct windows. However, such findings do not rule out packaging of the viral genome into a pre-assembled pro-capsid, since the late stage of infection provides sufficient time to accommodate multiple non-overlapping assembly and packaging events. In some experiments, the incorporation of labeled amino acids into empty particles could be detected slightly before (~1 h) incomplete and packaged particles could be detected (92, 111). This bias could represent the assembly of the first pro-capsids before any packaging occurs, an event that could suggest sequential capsid assembly and genome packaging. However, such slight differences should be interpreted with caution. For one thing, empty and incomplete particles may also co-purify with oligomeric hexon and/or hexon aggregates (111). This makes it difficult to determine whether this slight temporal bias results from early pro-capsid assembly or from the accumulation of hexon protein, which must precede assembly and packaging regardless of the packaging mechanism.

Many pulse-chase experiments utilized temperature-sensitive mutant viruses with conditional defects in packaging and/or maturation to explore the temporal relationship between different particle types. What is undeniably clear from studies of mutant viruses with temperature-sensitive defects in maturation is that packaged particles remain incomplete if maturation is prevented (110, 140, 155). However, since maturation occurs after the initial steps of viral genome packaging, this observation is consistent with both the sequential and concurrent models and does not favor one model over the other. Perhaps more pertinent to the packaging mechanism is the relationship between empty particles that lack viral DNA and mature particles. Under non-permissive conditions, mutants with defects in packaging accumulate only empty and incomplete particles. If empty capsids function as precursors to packaged virions, then restoring packaging might be expected to transform empty capsids into complete packaged virions. However, radio-labeling shows that empty and incomplete particles accumulated during infection at non-permissive temperatures are not packaged, following a shift to the permissive temperature, even when sufficient late proteins have accumulated and sufficient viral genomes have been synthesized (108, 136, 155, 157, 158). This may indicate that empty capsids are dead-end by-products rather than pro-capsid precursors. However, it has astutely been argued that empty capsids could be rendered incapable of packaging due to irreversible defects acquired during incubation at the non-permissive temperature and thus could represent artificial by-products that are not indicative of the true packaging mechanism (155). Indeed, in summarizing experiments analyzing the relationship between different types of particles accumulated during infection, Edvardsson et al. stated that it is “difficult to establish whether the capsids are built around the DNA or the DNA is introduced into preformed shells” (155).

In summary, attempts to establish the relationship between different types of AdV particles have not yet done enough to clarify whether capsid assembly and genome packaging are sequential or concurrent processes. We must be aware that if capsid assembly and genome packaging are coordinated processes linked in space and time, then it may be difficult to disrupt one of these processes without impacting the other. The solution may lie in the development of approaches that can determine the relationship between different particle types accumulated during infection, with greater temporal resolution than has been achievable to date.

## WHERE IS THE PORTAL?

Sequential packaging of a pre-formed pro-capsid requires a portal, an opening through which the viral genome can enter, along with associated packaging machinery that helps recognize the viral genome and provide the ATP-dependent motor-like function required to pump the genome into the pro-capsid (12, 13, 15–17). Typically, the viral portal and portal complex are present at a unique vertex of the pro-capsid prior to and during packaging. Following packaging, proteins of the portal complex are typically removed, and the portal is sealed, completing encapsidation (15, 17, 18, 159, 160). Members of the Herpesviruses and certain bacteriophages serve as prototype examples of DNA viruses that utilize a *bona fide* sequential packaging mechanism. In these prototype examples, the portal and portal complex have been identified *in situ*, and in many cases, the structures have been determined in detail (19, 21, 159, 161–163). In the case of HSV-1, the structure of the portal and packaging machinery in association with the viral genome has been resolved at high resolution by cryo-electron microscopy, providing great insight into how the viral genome is translocated into the particle (19). Such evidence leaves little doubt that these viruses package their genomes into pre-formed capsids.

It has been proposed that the AdV packaging complex comprising L1-52K, IVa2, IIIa, L4-33K, and L4-22K may fulfill a similar role to the portal complex of other DNA viruses (49, 52). However, evidence for an adenovirus portal complex is limited and circumstantial. Transmission electron microscopy combined with immuno-gold labeling demonstrates that putative packaging proteins IVa2 and L4-33K can be identified at or near a unique vertex of some viral particles (~ 10%–15%), as can the viral early proteins E4orf6 and DBP (123, 137). These findings have been presented in favor of sequential packaging mechanisms, in which E4orf6 functions as the portal, with L4-33K and IVa2 responsible for the ATP-dependent pumping of the viral genome into the pro-capsid (49, 123, 137). However, although the identification of packaging proteins proximal to a unique capsid vertex is consistent with the presence of an adenovirus portal complex, alternate explanations cannot be ruled out. For example, viral packaging proteins are reported to interact either directly or indirectly with viral genomes (49, 52, 121, 122). It is possible, therefore, that these proteins can be found proximal to the capsid due to their association with viral genomes that have either failed to package or are yet to complete the packaging process. Such a scenario could occur as the result of either sequential or concurrent packaging. This is particularly relevant, given that previous studies have demonstrated that viral genomes, which have initiated but failed to complete packaging, remain associated with viral particles even after purification (108, 118, 143, 144). It is also interesting to note that E4orf6 is not considered a canonical structural protein. E4orf6 is expressed as an early protein and contributes to host-cell reprogramming (164, 165). If E4orf6 also functions as part of the portal complex, it is unclear how this protein is repurposed to fulfill a role in viral genome packaging.

Studies of the structure of both complete and incomplete AdV particles provide high-resolution models of capsid structure and important insights into particle morphogenesis, including maturation (48, 55, 61, 62, 101). However, to the best of our knowledge, no portal or portal complex has yet been identified in the analysis of structural data. This lack of evidence is hard to ignore and will continue to stimulate debate on the likelihood that such a portal and portal complex exists. However, the absence of structural evidence does not necessarily constitute evidence for the absence of a portal. For example, it is possible that identification of the portal complex remains elusive because it is only present in unstable assembly intermediates that are difficult to isolate or because many computational approaches that leverage particle symmetry to resolve the structure of purified particles are not conducive to characterizing unique vertices. Definitively determining whether the viral particle contains a portal complex at some stage of assembly may require advanced Cryo-EM approaches capable of determining and resolving the structure of capsid vertices *in situ*, such as cryo-electron tomography.

If there is no portal and portal complex, then what is the function of the packaging sequence and packaging complex? Recognition of a packaging sequence by ATP-dependent packaging machinery is an important feature of sequential packaging (12, 16, 49). This fits nicely with the notion that capsid assembly can occur spontaneously, whereas translocation of the viral genome into the capsid against increasing internal pressure requires energy (166–169). However, this line of reasoning ignores the fact that the concurrent packaging mechanism may also require a packaging sequence and ATP-hydrolysis. Although some small DNA and RNA viruses package their genome without the use of specific packaging sequences or dedicated packaging ATPases, this is not the case for all. For example, specific sequences in the viral genome are also required for the concurrent packaging of RNA viruses, where they are thought to provide a docking site for viral proteins that initiate the assembly of the capsid around the genome (170). Furthermore, we now know that the use of ATPases for genome packaging is not exclusive to large DNA viruses that package their genome into pre-formed capsids. For example, even some very simple RNA viruses of plants that assemble their capsids around their genomic RNA are known to utilize the built-in ATPase activity of their capsid proteins for packaging (171). It is also possible that ATPases such as nucleic acid helicases and translocases contribute to packaging by altering the suitability of nucleoprotein complexes for packaging. For example, efficient packaging may require the removal of unwanted DNA-binding proteins or changes in nucleic acid structure. Clearly, there is still much to learn about the mechanisms by which the AdV packaging sequence and packaging proteins contribute to genome packaging. Elucidating detailed mechanistic insight into the function of specific packaging proteins will likely aid in resolving whether AdV packaging is sequential or concurrent.

## HOW IS CONDENSED DNA PACKAGED?

Another mystery of AdV packaging relates to the formation of the viral core, which comprises the condensed viral genome in association with viral core proteins (56, 172). Successful packaging of the AdV genome involves confinement of ~36,000 bp of dsDNA within the internal cavity of the capsid. This is an impressive feat when considering that this involves the compaction of a DNA molecule with an end-to-end length of approximately 12 µm into a space less than 100 nm in diameter (47, 173). Inside the viral particle, the viral genome is associated with small positively charged viral core proteins (µ, V, and VII) that are thought to shield the negative charge of the viral DNA and promote genome condensation (56, 57, 173). The viral genome and core proteins are organized as condensed “adenosomes” with different experimental approaches providing different estimates on the size and number of these clusters within the core (57, 79, 173–177). The network of interactions between the viral core and the capsid protein within the particle plays an important role in ensuring the timely disassembly of the viral particle and delivery of the viral genome into the nucleus (56, 64, 70, 173, 178–180). Interestingly, protein VII remains bound to the viral genome during and after the genome is imported into the nucleus. Following the nuclear import of invading viral genomes, a program of viral early gene expression is initiated. The early stage of infection coincides with a decrease in protein VII associated with the viral genome and the addition of cellular histones (79, 172, 181–183). Early gene expression enables replication of the viral genome, which requires interaction between the viral genome and the viral DNA replication machinery. Viral genome replication is a pre-requisite for late gene expression, suggesting that replication-dependent changes in viral chromatin may be required for viral late gene expression (58, 172). During the late stage of infection, the association of core proteins with viral genomes can be readily detected using approaches such as chromatin immunoprecipitation (ChIP) and isolation of proteins on nascent DNA (iPOND), the latter indicating that core proteins associate with viral genomes during or shortly after genome replication (58, 184). Packaged AdV particles contain the core proteins µ, V, and VII, but empty capsids do not, suggesting that core proteins are not present as part of the capsid and are likely incorporated into the particle in complex with the viral genome (127, 141). Collectively, these findings indicate that the viral genome exists in an organized state throughout the viral replicative cycle (172, 185).

The genomes of herpesviruses and phages are packaged as naked DNA that is pumped into the pre-formed capsid, resulting in compaction of the viral genome and significant internal pressure within the capsid shell (15, 17, 169, 186, 187). It is likely that the absence of organizing proteins, such as histones or histone-like proteins, facilitates this packaging mechanism, allowing the naked DNA substrate to be threaded through the small portal. In contrast, the available evidence indicates that the AdV genome is packaged as a nucleoprotein complex, rather than as naked DNA. Protein VII is the most abundant core protein within progeny particles and is thought to be the key player in condensation and organization of the viral genome (56, 172, 173, 188). When viral genomes associated with protein VII are isolated from viral particles, they exhibit a condensed organization resembling “beads on a string” that share similarities with cellular chromatin (189). Protein VII readily interacts with dsDNA and is alone sufficient to condense lampbrush chromosome DNA when expressed in Xenopus oocytes (190). The organization of viral DNA inside the particle has been more difficult to resolve, with EM studies suggesting a non-icosahedral core that is largely disordered and may exist as a liquid-like collection of condensed nucleoprotein bundles (47, 57, 64). The disruption of adenovirus particles by atomic force microscopy shows that the viral genome retains elements of this bundled organization as it is released from the viral particle (64, 173). It has long been assumed that positively charged core proteins facilitate packaging by countering the negative charge of viral DNA to promote viral genome condensation (13, 56). However, experiments with a conditional protein VII knock-out virus show that viral genomes can be packaged into particles in the absence of protein VII (188). Although dispensable for packaging, particles lacking protein VII exhibit defects in particle disassembly (173, 178, 188). This suggests that core proteins such as protein VII promote efficient delivery of the viral genome into the nucleus via their contribution to particle stability and disassembly (56, 64, 173). It seems likely, therefore, that the organization of the viral genome at the time of packaging is also crucial for infectivity of the resulting particle.

Collectively, data from the literature suggest that wild-type AdV packaging involves the packaging of a nucleoprotein complex rather than naked DNA. This poses an interesting conundrum: how is an organized viral genome packaged? After all, the existence of an adenovirus portal complex that possesses the complexity required to translocate a protein-bound genome or nucleoprotein core seems highly unlikely, as noted by others (4). Importantly, these challenges would be circumvented by concurrent assembly and packaging mechanisms in which viral core proteins associate with the viral genome prior to or during packaging. However, the concurrent packaging model raises its own questions. If protein VII is dispensable for packaging, then which viral (or host) proteins are responsible for condensing the viral genome and for initiating core formation and capsid assembly? Furthermore, how are core formation and capsid assembly coupled?

## THE ROLE OF THE L1-52K PACKAGING PROTEIN AND BIOMOLECULAR CONDENSATES IN PARTICLE PRODUCTION

The viral L1-52K protein is essential for genome packaging and interacts directly or indirectly with viral genomes, putative packaging proteins, and minor capsid protein IIIa (125, 127–129). Although the L1-52K protein co-purifies with empty and incomplete particles, it is not a component of mature virions, as it is proteolytically processed by the AdV protease and subsequently removed from the particle during particle maturation (61, 107, 108, 191, 192). This has led to the suggestion that L1-52K plays a key role in linking capsid assembly and genome packaging, perhaps by acting as a scaffold or “molecular Velcro” that physically links the viral genome to the assembled or assembling capsid (49, 142). However, the exact function of L1-52K in genome packaging remains somewhat enigmatic.

Recent advances in our understanding of subcellular organization suggest that many cytoplasmic and nuclear bodies are non-membranebound biomolecular condensates that form as a result of phase transitions, including liquid-liquid phase separation (193–195). This de-mixing of the cytoplasm or nucleoplasm enriches specific biomolecules, such as proteins and nucleic acids, within condensates while excluding others (194, 195). The organization of biomolecules into condensates can promote biological processes by concentrating factors to increase the frequency of interactions and reactions. Condensates can also regulate biological processes by sequestering factors and limiting their concentration in the wider cytoplasmic/nucleoplasmic phase (193, 195). We recently demonstrated that the AdV L1-52K packaging protein forms nuclear bodies that organize viral capsid proteins (141). These nuclear bodies exhibit many of the hallmarks of biomolecular condensates, including liquid-like properties such as the ability of condensates to fuse,and internal rearrangement of molecules within condensates (141, 196). Several lines of investigation support the notion that liquid-like viral nuclear bodies are critical for the assembly of packaged progeny particles in AdV-infected cells. The N-terminal intrinsically disordered region (IDR) of L1-52K is essential for phase separation *in vitro* and nuclear body formation in cells (141). This IDR has an interesting primary sequence, with a high arginine content and charged residues clustered into short stretches (8–15 amino acids), resulting in patches of sequential negative and positive charge along most of the IDR (141). This distribution of charged amino acids is consistent with a “spacers and stickers” model in which charged patches contribute to the dynamic multivalent interactions that promote phase separation (197–201). In contrast, the extreme N-terminal end of L1-52K lacks patches of significant charge, suggesting that this region may act as a “fluidizing entity” that provides competing interactions that regulate condensate properties (141). Consistent with these notions, mutation of 50% of the arginine residues to lysine, predicted to weaken IDR-IDR interactions (197–199, 202), attenuates phase separation *in vitro* and prevents nuclear body formation in cells (141). In contrast, mutation of the fluidizing entity by substitution of proline with alanine residues does not prevent phase separation but results in the formation of nuclear bodies that exhibit reduced dynamic exchange of L1-52K with the nucleoplasm compared with the wild-type protein (141). This loss of dynamic exchange is consistent with more solid-like properties (203–207). Both of these L1-52K mutants failed to complement progeny production when expressed *in trans* in cells infected with aanL1-52K-defective virus (141). We also showed that phosphorylation of serine residues within the N-terminal IDR (S28 and S74) maintains the liquid-like properties of nuclear bodies and promotes the production of packaged progeny particles (196). These findings highlight the importance of the IDR and evidence of the functional contribution of nuclear bodies to packaging. Interestingly, mutations outside of the N-terminal IDR of L1-52K can also impact nuclear body function. The mutant ts369 has a double amino acid substitution (QL/GP) within the C-terminal region (positions 334–335) of L1-52K and fails to package its genome at the non-permissive temperature of 39.5°C (108). Under these conditions, the viral minor capsid protein IIIa, an interaction partner of L1-52K and a key determinant of successful packaging, fails to localize to nuclear bodies in infected cells (141). This suggests that packaging requires the recruitment of this key component into nuclear bodies. Collectively, these findings suggest that the assembly of packaged progeny particles requires the organization of L1-52K and capsid proteins into nuclear bodies with specific properties. The proximity of these nuclear bodies to viral replication compartments and sites of genome replication, coupled with the requirement for active genome replication, suggest that condensate function is linked to the provision of newly replicated genomes released from viral replication compartments. Our working model proposes that viral nuclear bodies function as storage sites that organize L1-52K and capsid structural proteins, concentrating these proteins while ensuring that they remain conducive to assembly. This regulates the assembly of capsid proteins, such that capsid assembly is coordinated with the provision of newly replicated genomes required for packaging (141). In turn, this implies that capsid assembly and genome packaging are intimately linked in space and time, a notion that fits well with the concurrent model of assembly and packaging.

The organization of L1-52K and capsid proteins into biomolecular condensates may also explain why a small proportion of dead-end empty capsids accumulate during infection. The formation of biomolecular condensates is concentration dependent and results in a dense phase in which condensate proteins are enriched (nuclear bodies) and a light phase (nucleoplasm) in which a low concentration of condensate protein is maintained (193–195). This may represent an elegant strategy by which capsid proteins can be accumulated to high levels in the nucleus while limiting dead-end aggregation of capsid proteins in the nucleoplasm. If capsid assembly is spontaneous, then it would be expected that L1–52K and capsid proteins present at low levels in the nucleoplasm should assemble into a limited number of capsids independent of the provision of viral genomes. This could explain why some empty capsids accumulate during infection. Since condensate formation is concentration-dependent, and viral late proteins are synthesized *de novo*, an initial period of late protein accumulation in the nucleoplasm must precede condensate formation. This phenomenon could explain the slight bias toward the labeling of empty capsids during the very beginning of the late phase in radio-labeling experiments (92, 111).

Although the discovery of a role for condensates in assembly and packaging provides novel insight into how these processes are coordinated, and a fresh perspective from which to view old challenges, several key questions remain. For example, do L1-52K protein nuclear bodies function by sequestering capsid proteins to regulate the timing and rate of capsid assembly with the timing and rate of genome replication? This could represent a very elegant strategy by which viral capsid proteins can accumulate at very high abundance while limiting capsid protein aggregation that could compromise successful capsid assembly. Is it possible that these nuclear bodies also contribute to packaging by providing a network of dynamic protein-protein or protein-DNA interactions or a physical environment conducive to genome packaging? Importantly, what are the molecular events that initiate and couple genome packaging and capsid assembly? It has been proposed that L1-52K could act as a scaffold or molecular glue that physically links the assembling capsid and nascent core (64). If this is the case, then could L1-52K play a more direct role in the initiation of an assembly intermediate that couples genome packaging and capsid assembly? Naturally, understanding whether capsid assembly and genome packaging are sequential or concurrent processes will likely require elucidation of the molecular events that take place when viral genomes, L1-52K, and structural proteins meet.

## INVESTIGATING VIRAL ASSEMBLY AND GENOME PACKAGING *IN SITU*

One major barrier to understanding the specific mechanistic events that take place during viral genome packaging is the lack of experimental approaches capable of characterizing the processes of capsid assembly and genome packaging as they happen. Recently, the field has applied several new technical approaches to visualize capsid assembly and genome packaging *in situ*. Condezo et al. employed electron microscopy to look for putative packaging/assembly intermediates in AdV-infected cells (142). They identified intermediates that appear to represent different stages in viral morphogenesis, including viral cores and partially encapsidated cores that may indicate genome packaging that is in process. It is of note that the model of adenovirus assembly/packaging proposed by Condezo et al. shares some similarities with recent work that characterized the assembly and packaging of bluetongue virus (BTV), a segmented dsRNA virus. Cryo-electron microscopy of purified assembly intermediates and electron tomography of the infected cells identified 11 different sub-types of BTV particles representing different stages in assembly and packaging (208). The authors propose a hybrid model of BTV packaging in which core proteins first co-assemble with viral genomic RNA to package the genome and form a sub-core around which the full core and capsid are built. This model also proposes that a proportion of particles are built around pre-cores that have failed to incorporate genomic RNA, providing an explanation for how failed packaging at this early stage can result in the formation of dead-end empty capsids. Broadly speaking, this revised model of BTV packaging and assembly illustrates how viral assembly and packaging may proceed via multiple stages in a stepwise fashion and may exhibit a complexity not fully captured by canonical packaging models.

**Fig 2:**
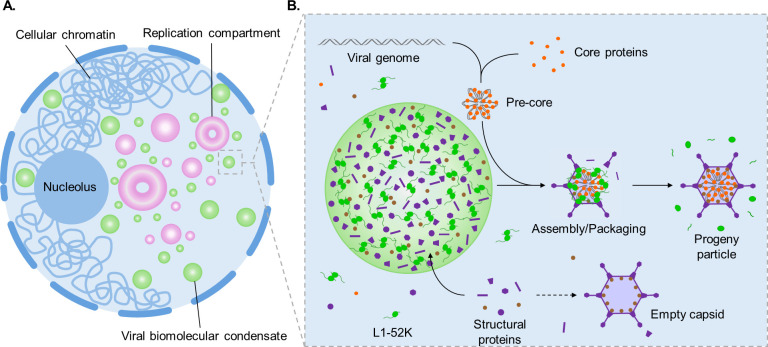
Model of coordinated assembly and packaging in the adenovirus-infected nucleus. (A) Viral replication compartments (pink) function as organizing hubs of viral genome replication, which provides viral genomes for packaging into progeny particles. Viral biomolecular condensates (green) form during the late stage of infection and are required for the assembly of packaged progeny particles. (B) The L1-52K packaging protein and capsid proteins including but not limited to IIIa, hexon, and fiber are enriched in viral biomolecular condensates. A nascent viral genome associates with viral core proteins, and the formation of the pre-core begins. Packaging occurs at the intersection of these pathways and involves the assembly of the capsid around the pre-core. The L1-52K facilitates this process by coupling the pre-core to the assembling capsid. Incorporation of the viral protease and its co-factors results in the proteolytic processing of structural proteins either during or after packaging, yielding the mature packaged particle. Empty capsids are assembled from capsid proteins in the nucleoplasm and are dead-end byproducts that result from capsid assembly that is not coordinated with the provision of viral genomes.

Condezo et al. identified AdV packaging intermediates and incomplete particles at the sites of genome replication (142). The authors suggest that viral genomes are condensed as the genome is replicated or immediately after and that the capsid assembles around the nascent core. This model is in keeping with previous observations that packaging requires active genome replication (135, 136). A recent study by Gomez-Gonzalez et al. also supports a concurrent model of coordinated replication, core formation, and capsid assembly (209). The authors utilized stimulated emission depletion microscopy, single object tracking, fluorescence recovery after photobleaching, and correlative fluorescence-electron microscopy to explore the spatial organization of AdV progeny production. They combined these techniques with dual genome labeling and click chemistry to track temporally distinct sub-populations of viral genomes post-synthesis. The authors identified nuclear puncta that emerged from viral replication compartments during the late stage of infection. These puncta were positive for both newly synthesized viral genomes and core protein V, suggesting that viral core formation and genome replication are intimately linked in space and time. These authors propose a stepwise assembly model, in which core formation begins prior to interaction with L1-52K and capsid proteins and encapsidation of the core (209). Interestingly, Condezo et al. demonstrated that viral cores accumulate in infected cells when packaging is attenuated, suggesting that core formation can occur independently from capsid assembly (142). Collectively, these findings suggest a spatially and temporally coordinated model of stepwise assembly in which the genome first interacts with core proteins to form a pre-core that is then encapsidated (Fig. 2). These advances further challenge the dogmatic sequential model in favor of concurrent capsid assembly and genome packaging.

## ARE ALL VIRAL GENOMES CREATED EQUAL?

The apparent coordination of genome replication and genome packaging in space and time suggests that viral packaging utilizes newly replicated viral genomes. This raises questions that seem almost paradoxical when considering the prevailing dogma that assumes all viral genomes to be functionally equivalent. If only nascent genomes are packaged, as suggested by recent models of adenovirus assembly and packaging (141, 142, 209), then what happens to the “old” genomes? Indeed, is it possible that only a specific subset of genomes is destined to be packaged? This would be consistent with the supposition that the number of viral genomes accumulated in infected cells exceeds the number of genomes that are packaged. Multiple studies have now corroborated the observation that “new” viral genomes are synthesized at VRCs before moving away (209–214). This demonstrates that although some nascent genomes must be retained at VRCs for further rounds of replication or are recruited for packaging, others leave and presumably fulfill alternate functions. The tracking of viral genomes post-replication also shows that viral genomes accumulate in late-phase compartments designated as virus-induced post-replicative (ViPR) bodies (212–214). It was proposed that viral genomes accumulate in ViPR bodies before being processed for packaging, since ViPR bodies are enriched for cellular chromatin modifiers that could play a role in removing histones from viral genomes. This could explain why cellular histones are present on viral genomes in the infected cell but absent in packaged particles (172). However, this model does not explain the requirement of active genome replication for packaging and is inconsistent with recent findings, which suggest that genome replication and packaging are intimately linked in space and time (135, 136, 141, 142, 209).

Speculatively, this seemingly paradoxical situation might be resolved if we reject the assumption that all viral genomes are functionally equivalent. Is it possible that distinct populations of viral genomes contribute to either late gene expression or packaging but not to both? Such a strategy may represent an elegant way of negotiating the requirement of viral genomes for multiple competing processes and a way of protecting genomes destined for packaging from the DNA damage and mutation that are associated with a high transcriptional burden (215, 216). If viral genomes are packaged immediately after their synthesis, before histones can be added, then there may be no need to remove histones for packaging. It follows that accumulated genomes associated with cellular histones would not be required for packaging but instead may represent a pool of genomes utilized for transcription. This would be consistent with reports that suggest only newly replicated viral genomes are packaged (141, 142, 209). Currently, many of the techniques applied to study viral processes rely on the analysis of viral genomes isolated from cells in bulk. Interrogation of the hypothesis that different populations of genomes fulfill distinct functions will likely require the development of techniques that can isolate specific sub-sets of genomes or more approaches that can assess genome function at single-molecule resolution.

## CONCLUSIONS AND FUTURE PERSPECTIVES

Over the last 50 years, the study of AdV capsid assembly and genome packaging has provided insights into these critical viral processes. To date, the majority of approaches have relied on the characterization of viral particles purified from infected cells. The isolation and comparison of viral particles from cells infected with wild-type AdV or mutants lacking specific late proteins or the viral packaging sequence have helped identify key requirements for genome packaging. In combination with classical molecular biology techniques, these observations substantiate the importance of the AdV packaging sequence and putative packaging proteins essential for genome packaging. These studies have identified the key players and laid the groundwork for understanding adenovirus assembly and packaging. However, in our opinion, these experiments have not yet fully resolved the mechanism of genome packaging. Recent studies suggest that capsid assembly, genome replication, and genome packaging are intimately linked, stimulating renewed interest in the concurrent packaging model in which the capsid shell is assembled around the viral genome. The available evidence accrued over several decades seems to fit better with this concurrent model. However, the concurrent packaging model presents its own mysteries, which must be resolved if we are to advance our understanding of the AdV packaging mechanism. For example, how are components of assembly and packaging recruited in a coordinated fashion, how is the genome condensed for packaging, and what critical function is fulfilled by the packaging proteins and the packaging sequence?

Understanding better the process of AdV packaging will likely require experimental approaches capable of characterizing individual steps in the process as they happen. We call for further investigation into AdV packaging and recommend that important challenges in resolving the AdV packaging mechanism are faced with an open mind, since viral assembly and packaging mechanisms may be more complex and diverse than previously appreciated. Key features of AdV packaging have been interpreted in line with existing models of assembly and packaging that are based on the characterization of relatively few prototype DNA viruses. It is possible that broadly applied sequential or concurrent models may fail to accommodate fully specific mechanistic features or stages of AdV packaging.

For many decades, the study of AdV biology has pioneered some of the most important findings in biochemistry, cell biology, and virology. We believe that the study of AdV may yet lead to advances in our understanding of DNA packaging, advances that could be broadly applicable to the host cell genome, other viruses, and the design and production of next-generation viral vectors for therapeutic gene delivery. A comprehensive understanding of viral assembly and genome packaging could enable novel approaches in vector engineering and production. For example, cell-free assembly and packaging of viral vectors offer the potential for maximized coding capacity and modification. However, such high-potential approaches have so far remained elusive. As we look to the future, we should also consider how viral assembly, packaging, and gene-delivery strategies can inform and inspire the next generation of synthetic gene-delivery vectors. Viruses are the true masters of genome packaging and gene delivery, and AdV serves as a prime example. There may still be much that AdV can teach us, lessons that may be highly valuable in the modern world of gene therapy, vaccines, and oncolytic therapies.
